# Integrated management strategies increased silage maize yield and quality with lower nitrogen losses in cold regions

**DOI:** 10.3389/fpls.2024.1434926

**Published:** 2024-07-22

**Authors:** Changqing Li, Bingxin Tong, Mengyang Jia, Huasen Xu, Jiqing Wang, Zhimei Sun

**Affiliations:** ^1^ College of Resources and Environmental Sciences, Hebei Agricultural University, Baoding, China; ^2^ Department of Soil and Fertilizer Management, Zhangjiakou Soil and Fertilizer Station of Hebei Province, Zhangjiakou, China; ^3^ College of Agriculture and Forestry Sciences, Hebei North University, Zhangjiakou, China

**Keywords:** silage maize, variety and density, nitrogen fertilizer management, yield and quality, nitrogen balance integrated management strategy

## Abstract

**Introduction:**

High-yield and high-quality production of silage maize in cold regions is crucial for ensuring the sustainable development of livestock industry.

**Methods:**

This study first conducted an experiment to select the optimized silage maize varieties and densities using a split-plot design. The tested maize varieties were Xuntian 3171, Xuntian 16, Xunqing 858, and Fengtian 12, with each variety planted at densities of 67,500, 79,500, and 90,000 plants ha^-1^. Following the variety and density selection, another experiment on optimizing nitrogen management for silage maize was carried out using a completely randomized design: no nitrogen fertilizer (T1), applying urea-N 320 kg ha^-1^ (T2), applying urea-N 240 kg ha^-1^ (T3), applying polymer-coated urea-N 240 kg N ha^-1^ (T4), and ratios of polymer-coated urea-N to urea-N at 9:1 (T5), 8:2 (T6), 7:3 (T7), and 6:4 (T8). T5-T8 all applied 240 kg N ha^-1^. The yield and quality of silage maize, nitrogen use efficiency and balance, and economic benefits were evaluated.

**Results:**

Results showed that Xunqing 858 had significantly higher plant height (8.7%-22.6% taller than the other three varieties) and leaf area (30.9% larger than Xuntian 3171), resulting in yield 11.5%-51.6% higher than the other three varieties. All varieties achieved maximum yields at a planting density of 79,500 plants ha^-1^. Integrated management strategy 7 (T7: Xunqing 858, 79,500 plants ha^-1^, polymer-coated urea-N to urea-N ratio of 7:3) achieved the highest yield of 73.1 t ha^-1^, a 6.1%-58.1% increase over other treatments. This strategy also produced the highest crude protein (11.1%) and starch (19.1%) contents, and the lowest neutral detergent fiber content (50.6%), with economic benefits improved by 10.3%-97.8% compared to other strategies. Additionally, T7 improved nitrogen use efficiency by 15.4%-94.5%, reduced soil nitrate leaching by 4.4%-36.5%, and decreased nitrogen surplus by 7.0%-46.6%.

**Conclusion and discussion:**

Comprehensive analysis revealed that the integrated management strategy 7 significantly improved silage maize yield and quality in cold regions while enhancing nitrogen use efficiency and reducing the risk of nitrate leaching, aligning with green agriculture development requirements. These findings will provide vital theoretical insights and practical guidance for high-yield and high-quality silage maize production in cold regions worldwide.

## Graphical Abstract

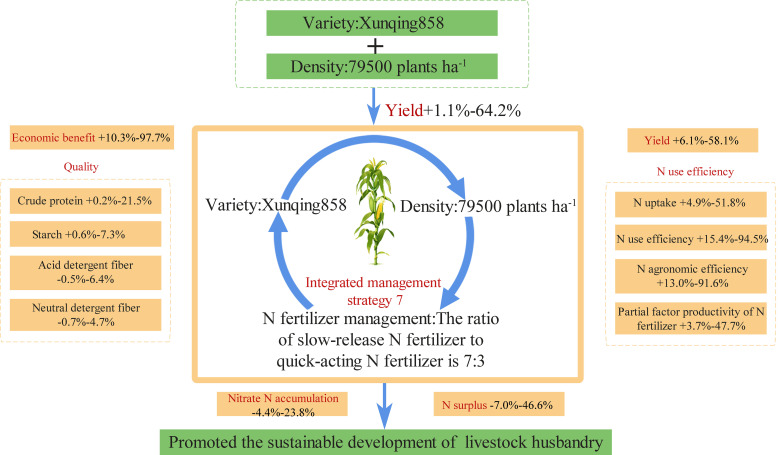

## 1 Introduction

Economic growth and improved living standards had led to a substantial increase in the consumption of dairy and meat products, and this trend continues to increase in the future (Komarek et al., 2021; Du et al., 2023). For instance, from 2013 to 2022, the per capita consumption of meat and dairy products increased by 35.16% and 30.15%, respectively (China Statistical Yearbook, 2014, 2023). However, the sustainable development of livestock husbandry is under threat due to the degradation of natural grasslands caused by overgrazing and drastic climate change, which in turn impacts the meat and milk supply (Wheeler and von Braun, 2013; Niu et al., 2019; Zhao et al., 2019). Silage maize is becoming a good substitute for natural forage because of its economic efficiency (low cost) and excellent nutritional properties (rich in nutrition, highly palatable and digestible) (Guyader et al., 2018; Bilal et al., 2021; Galeano et al., 2021). But now, silage maize production is facing great challenges, including the lack of superior varieties, inappropriate planting density, and suboptimal water and fertilizer management, leading to unstable and lower yields, poor feed quality, and serious environmental pollution. Therefore, it is imperative to develop high-yield, high-quality, cost-effective and eco-friendly silage maize production technology.

Integrated Soil-Crop System Management (ISSM) offers a practical solution for achieving synergistic improvements in yield, quality, and environmental benefits in crop production systems. By utilizing appropriate crop varieties, optimal sowing dates, suitable planting densities, and advanced nutrient and water management strategies, ISSM restructures the entire production process to align with local environmental conditions (Chen et al., 2011). This approach has demonstrated substantially increase in summer maize grain yield, ranging from 33.0%-86.8%, while simultaneously reducing nitrogen losses by 39.0% to 88.9% (Chen et al., 2014; Liu et al., 2018). However, the overall scheme suitable for silage maize that ensures high yield, superior quality and environmental sustainability remains unclear.

High-latitude and high-altitude regions generally serve as crucial bases for the development of the livestock industry. However, these regions face severe challenges, including year-round drought and cold, short frost-free periods, and soil with poor capabilities of retaining water and fertilizer. These factors make it difficult to ensure the yield and quality of silage maize, as highlighted by Yang et al. (2019) and Bai et al. (2022). Crop yield relies on complex interactions between genotypes, environmental factors (including climate and soil conditions), and agricultural management. Among these, the yield-increasing potential of genotype is an important aspect. Liu et al. (2021) showed that the contribution of variety improvement to grain yield was 111.4 kg ha^-1^ year^-1^. Research by Kumar et al. (2022) has shown that planting early-maturing varieties of silage maize in high-latitude regions could increase dry matter content, starch content, and organic matter digestibility. However, the current silage maize varieties are generally chaotic and miscellaneous, and the adaptability and resistance of different varieties have obvious differences. Meanwhile, different silage maize varieties have different optimal planting densities because of their different plant types. Evidence points towards plant density as one of the critical indicators in explaining maize yield booms in the USA and other parts of the world (Duvick, 2005). In North America, optimum plant density increased at a rate of 700 plants per hectare per year during 1987-2016 (Assefa et al., 2018). Furthermore, another study demonstrated that in high-altitude regions, the optimum plant density for silage maize production can be beyond 138900 plants ha^-1^ (Fallah and Tadayyon, 2010). However, under the condition that the density of maize hybrids continues to increase, the light conditions will deteriorate and the yield will decrease. It can be seen that the yield can be effectively improved on the basis of optimizing varieties and cooperating with the best planting density. Thus, the selection of varieties and their optimized density is of great significance to ensure the yield and quality of silage maize in this region. Additionally, sufficient nutrients are required because of higher biomass of silage maize during the whole growth period. In this case, over-fertilization is becoming common in actual production. According to our survey, the conventional nitrogen application rate of farmers in northwest Hebei province of China is as high as 320 kg ha^-1^. Relevant research showed that the nitrate content of groundwater in this region has reached 20~30 mg L^-1^, which is 2~3 times that of the American standard for drinking water (Ru et al., 2013). Excessive synthetic N fertilization has resulted in severe soil degradation and environmental pollution in agricultural system (Wu et al., 2024). Consequently, there is an urgent need to develop an integrated management strategy suited for silage maize production in cold regions.

In conclusion, hypothesizing that the integrated management strategy can improve the yield and quality of silage maize and reduce soil nitrogen loss in cold regions, the objectives of this study are as follows: (1) assessing the influence of the integrated management strategy on the yield and quality of silage maize in these cold regions; (2) elucidating the effects of integrated management strategy on nitrogen use efficiency, nitrogen balance, and economic benefits. The findings of this study will offer vital theoretical insights and practical guidance for enhancing yield and economic returns, promoting efficient resource utilization, and fostering the sustainable development of the livestock industry in these specified regions.

## 2 Materials and methods

### 2.1 Experimental site

**Figure 1:**
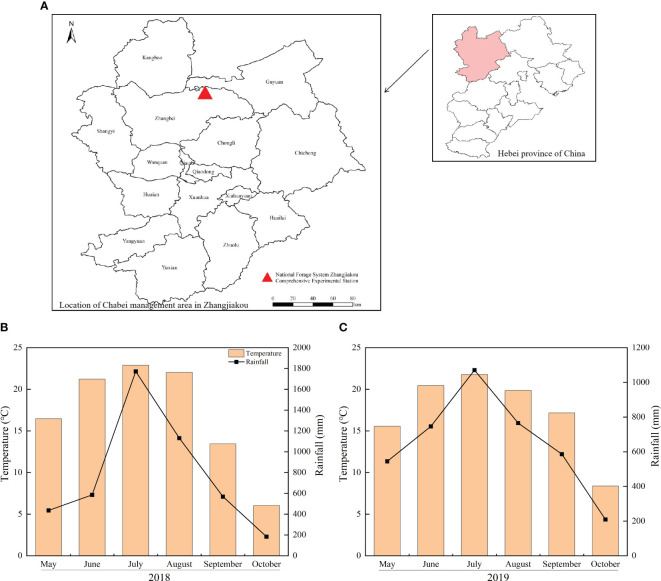
Experimental site and climate map. **(A–C)** represent experimental site, temperature and precipitation in 2018 and temperature and precipitation in 2019, respectively.

The experimental site (41°28’24” N, 115°1’3” E, altitude 1450 m) for this study was situated at the Comprehensive Experimental Station of the National Forage System in Zhangjiakou, as depicted in **Figure 1A**. This area was typically a continental monsoon climate with a frost-free period lasting 100 days. The tested soil was classified as chestnut soil. The chemical properties of tested soil in 0-20 cm layer were recorded as follows: Organic matter, 24.6 g kg^-1^, alkali-hydrolyzable N, 50.1 mg kg^-1^, available P, 13.2 mg kg^-1^, available K, 130.2 mg kg^-1^, and pH, 8.0. The soil bulk density in 0-20 cm, 20-40 cm, 40-60 cm, 60-80 cm, and 80-100 cm layer was 1.4, 1.5, 1.6, 1.5, and 1.5 g cm^-3^, respectively. The total precipitation amounts were 4675.3 mm and 3921.9 mm, and the average temperature was 17.0°C and 17.2°C during the growing seasons in 2018 and 2019 (shown in **Figures 1B**, **C**).

### 2.2 Experimental design

#### 2.2.1 Selection of best silage maize variety and planting density

This experiment involved two factors, viz., maize variety and planting density. The tested four silage maize varieties as main factor included Xuntian 3171, Xuntian 16, Xunqing 858, and Fengtian 12. Three planting densities as sub-factor comprised 67,500 (A1), 79,500 (A2), and 90,000 (A3) plants ha^-1^. Each treatment was repeated in triplicate, with each plot measuring 7 m by 4 m, totaling 28 square meters. The maize was sown on May 10^th^, 2018, and harvested on October 8^th^ of the same year. The application amount of urea (N, 46%), calcium superphosphate (P_2_O_5_, 18%) and potassium sulfate (K_2_O, 50%) were 320 kg N ha^-1^, 90 kg P_2_O_5_ ha^-1^, and 90 kg K_2_O ha^-1^, respectively. Of this, one-third of the urea was used as base fertilizer (applied before sowing), and two-thirds as topdressing (applied at the big trumpet period). Both calcium superphosphate and potassium sulfate were applied as base fertilizers. All other management strategies were consistent with those used in typical high-yield fields.

#### 2.2.2 Optimization of nitrogen management in silage maize planting system

Expanding upon the selection of the optimal variety (Xunqing 858) and density (79,500 plants ha^-1^), an experiment was conducted to optimize nitrogen fertilizer management. Eight treatments were arranged in a completely randomized design and repeated in triplicate, which included: (1) no application of nitrogen fertilizer (as control, T1); (2) applying urea-N 320 kg ha^-1^ as traditional fertilization according with that in farmers’ practice (as integrated management 2, T2); (3) applying urea-N 240 kg ha^-1^ (as integrated management 3, T3); (4) applying polymer-coated urea-N 240 kg N ha^-1^ (as integrated management 4, T4); (5) ratio of polymer-coated urea-N to urea-N at 9:1 (as integrated management 5, T5); (6) ratio of polymer-coated urea-N to urea-N at 8:2 (as integrated management 6, T6); (7) ratio of polymer-coated urea-N to urea-N at 7:3 (as integrated management 7, T7); (8) ratio of polymer-coated urea-N to urea-N at 6:4 (as integrated management 8, T8). Treatments T5, T6, T7, and T8 all applied 240 kg N ha^-1^, and all the above treatments contained the same amount of 90 kg P_2_O_5_ ha^-1^ and 90 kg K_2_O ha^-1^ as basal fertilizers according to the conventional recommended fertilizer rate in the local region. 40% N was applied before planting, and 60% N was applied during big trumpet period. The length and width of each experimental plot was 7 m and 8 m, respectively. Seeds were sown on May 19^th^ and harvested on October 2^nd^ in 2019. All other field management practices, such as weed control and irrigation, were consistent with those used in typical high-yield fields.

### 2.3 Sample collection and measurement

The agronomic traits of silage maize were measured at the milk maturity stage. For this purpose, 10 consecutive maize plants were selected from each plot to determine their plant height, stem diameter, and maximum leaf area. The maximum leaf area was calculated according to leaf length × leaf width × 0.75 (Zhao et al., 2020; Sezer et al., 2021).

For the determination of yield and quality of silage maize, two square meters from each plot were randomly chosen at the milk maturity stage, and the silage maize was cut at a height of 3 cm above the ground. The harvested maize was weighed on site, and recorded. Approximately 1 kg of the mixed sample of chopped was randomly taken for precise weight recording. All plant samples were first oven-dried at 105°C for 30 min and subsequently at 70°C until reaching constant weight. The dried samples were ground. Measurements were then conducted for crude protein content (using the kjeldahl method), acid detergent fiber content (ADF, using acid detergent method), neutral detergent fiber content (NDF, using neutral detergent method), crude starch content (using rotation method), and total nitrogen content (using the kjeldahl method), as detailed by Zeng et al. (2022).

Soil samples from 0-20 cm soil layer were collected and then air-dried before sowing to determine the basic physico-chemical properties. Soil organic matter content and alkali-hydrolyzable nitrogen were determined using the potassium dichromate-external heating method and alkali-dispersion method, respectively. Available phosphorus and potassium were determined using vanadium molybdenum blue colorimetry method and flame photometry method, respectively. Soil pH was measured using the glass electrode method with a water-to-soil ratio of 2.5:1. At the milk maturity stage of silage maize, post-harvest soil samples were collected randomly from three sites in each plot using an auger (inner diameter 2.0 cm) to a depth of 100 cm at 20cm intervals. The three samples from each plot at the same depth were thoroughly mixed to form one composite sample per depth and then transported in coolers on ice to laboratory. Fresh soil samples were immediately analyzed for nitrate nitrogen using ultraviolet spectrophotometry, and soil water content was determined after drying at 105°C for 24 h. Soil bulk density of each layer was measured using the ring knife method (Bao, 2000).

### 2.4 Statistical analysis and relevant calculation

The significance among treatments was analyzed by analysis of variance (ANOVA) using the SPSS 13.0 software (SPSS Inc., Chicago, IL, USA). For multiple comparisons, we used Least Significant Differences (LSD) at the 5% level. Data analysis in this study was conducted using Microsoft Excel for computation and OriginPro 2022 for graphical representation.

The formulas used for various calculations are as follows, in line with the methodology described by Zhang et al. (2008):


$$\begin{array}{c} \text{Plant nitrogen uptake }\left({\text{kg ha}}^{-1}\right)\\ =\text{plant dry matter }\left({\text{kg ha}}^{-1}\right)\times \text{plant nitrogen content }\left(\%\right) \end{array}$$

$$\begin{array}{c} \text{Nitrogen use efficiency }\left(\text{NUE},\;\%\right)\\ =(\text{ plant nitrogen uptake from treatments with N fertilizer}\\ -\text{plant nitrogen uptake in treatments without N fertilizer})\;\left({\text{kg ha}}^{-1}\right)\\ /\text{the application rate of N fertilizer }\left({\text{kg ha}}^{-1}\right)\times 100 \end{array}$$

$$\begin{array}{c} \text{Nitrogen agronomic efficiency }\left(\text{NAE},{\text{ kg kg}}^{-1}\right)\\ =(\text{the grain yield from treatments with N fertilizer}\\ -\text{the grain yield in treatments without N fertilizer})\;\left({\text{kg ha}}^{-1}\right)\\ /\text{the application rate of N fertilizer }\left({\text{kg ha}}^{-1}\right) \end{array}$$

$$\begin{array}{c} \text{Partial factor productivity of nitrogen fertilizer }\left({\text{PFP}}_{\text{N}},{\text{ kg kg}}^{-1}\right)\\ =\text{the grain yield from treatments with N fertilizer }\left({\text{kg ha}}^{-1}\right)\\ /\text{the application rate of N fertilizer }\left({\text{kg ha}}^{-1}\right) \end{array}$$

$$\begin{array}{c} \text{Nitrate nitrogen accumulation }\left({\text{kg ha}}^{-1}\right)\\ =(\text{the soil nitrate content in the corresponding soil layer }({\text{mg kg}}^{-1})\\ \times \text{the soil layer thickness }\left(\text{cm}\right)\times \text{the soil bulk density }\left({\text{g cm}}^{-3}\right))/10 \end{array}$$

$$\begin{array}{c} \text{Nitrogen surplus }\left({\text{kg ha}}^{-1}\right)\\ =(\text{Chemical-N}+\text{Irrigation-N}+\text{Atmospheric deposition-N}\\ +\text{Mineralized-N})\;\left({\text{kg ha}}^{-1}\right)-(\text{Plant remove N}+0-40{\text{ cm NO}}_{3}^{\;-}\text{-N})\\ \;\left({\text{kg ha}}^{-1}\right) \end{array}$$

Note: Chemical N refers to nitrogen brought by fertilizer input. The irrigation-N and atmospheric deposition-N are based on the research findings of Ju et al. (2006) and Liu et al. (2013), respectively. Mineralized-N refers to the amount of nitrogen absorbed by plants under the condition of no fertilization.


$$\text{Output value }\left(\${\text{ ha}}^{-1}\right)=\text{yield }\left({\text{t ha}}^{-1}\right)\times \text{unit price }\left(\${\text{ t}}^{-1}\right)$$

$$\begin{array}{c} \text{Economic benefit }\left(\${\text{ ha}}^{-1}\right)=\text{output value }\left(\${\text{ ha}}^{-1}\right)\\ -\text{fertilizer cost }\left(\${\text{ ha}}^{-1}\right)-\text{other costs }\left(\${\text{ ha}}^{-1}\right) \end{array}$$

Note: The expenses of fertilizers, the price of fresh grass, and other costs including irrigation, labor, pesticides, and machinery were calculated in accordance with the prevailing market prices of 2019.

To comprehensively evaluate the effects of different management strategies, the data pertaining to yield, crude protein, nitrogen uptake, nitrogen use efficiency, nitrogen surplus, and economic benefit were all standardized (Wang et al., 2020) according to the formula $\frac{\text{x}-\bar{\text{x}}}{\text{Std}}$ . Among this formula, $\bar{\text{x}}$ stands for average and Std stands for standard deviation.

## 3 Results

### 3.1 Effect of variety and density on yield and agronomic traits of silage maize

**Figure 2:**
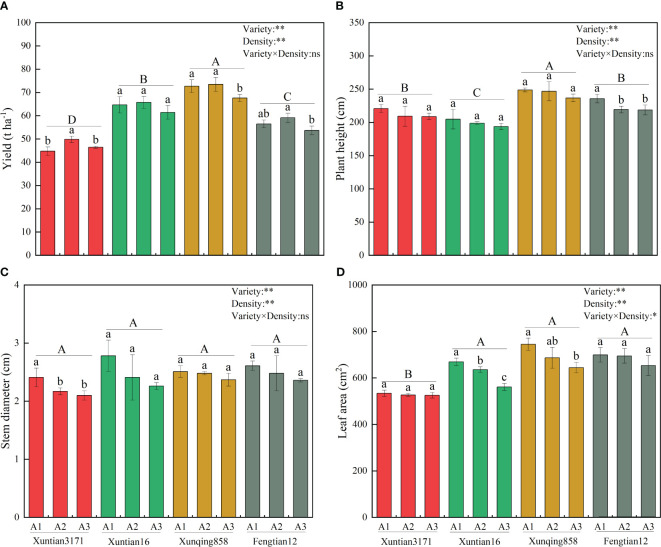
Effect of variety and density on yield and agronomic traits of silage maize. Panels **(A-D)** represent yield, plant height, stem diameter, and leaf area, respectively. A1, A2 and A3 represent planting densities of 67,500, 79,500 and 90,000 plants ha^-1^, respectively. Different lowercase letters indicate that different densities of the same variety have significant differences at the level of 0.05, and different uppercase letters indicate that different varieties have significant differences at the level of 0.05. ** and * represent significance at the 0.01 and 0.05 probability level, respectively, and ns represents no significance at the 0.05 probability level. No application of nitrogen fertilizer (as control, T1); Applying urea-N 320 kg ha^-1^ according with that in farmers’ practice (as integrated management 2, T2); Applying urea-N 240 kg ha^-1^ (as integrated management 3, T3); Applying polymer-coated urea-N 240 kg N ha^-1^ (as integrated management 4, T4); Ratio of polymer-coated urea-N to urea-N at 9:1 (as integrated management 5, T5); Ratio of polymer-coated urea-N to urea-N at 8:2 (as integrated management 6, T6); Ratio of polymer-coated urea-N to urea-N at 7:3 (as integrated management 7, T7); Ratio of polymer-coated urea-N to urea-N at 6:4 (as integrated management 8, T8).

The yield, plant height, stem diameter, and leaf area of silage maize were significantly influenced by both variety and density. However, the interaction between these two factors only has a significant effect on leaf area, as depicted in **Figures 2A–D**. Among the four examined varieties, Xunqing 858 exhibited a significantly higher yield by 11.5%-51.6% than the other varieties. Moreover, the yield of all varieties initially increased with planting density, but subsequently decreased, and reaching its peak value at a planting density of A2 (79,500 plants ha^-1^) (**Figure 2A**).

Concerning agronomic traits, it was observed that the plant height, stem diameter, and leaf area of silage maize progressively diminished as the planting density increased, as illustrated in **Figures 2B–D**. The stem diameter showed no significant differences among the four varieties. Xunqing 858 was distinguished by having the tallest plants and the largest leaf area. In comparison to the other varieties, the plant height of Xunqing 858 was significantly greater by 8.7%-22.6%, and its leaf area was substantially larger by 30.9% than that of Xuntian 3171.

### 3.2 Effect of integrated management strategies on yield and quality of silage maize

**Figure 3:**
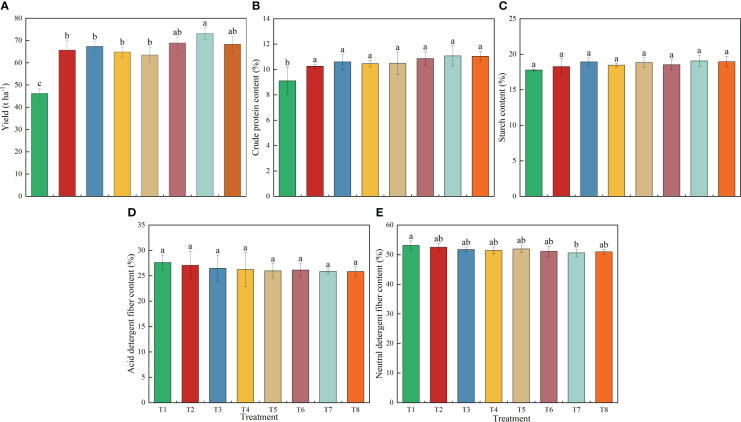
Effect of different management strategies on yield and quality of silage maize. Panels **(A-E)** represent yield, crude protein, starch, acid detergent fiber and neutral detergent fiber, respectively. Different lowercase letters indicate the significant differences at the level of 0.05. No application of nitrogen fertilizer (as control, T1); Applying urea-N 320 kg ha^-1^ according with that in farmers’ practice (as integrated management 2, T2); Applying urea-N 240 kg ha^-1^ (as integrated management 3, T3); Applying polymer-coated urea-N 240 kg N ha^-1^ (as integrated management 4, T4); Ratio of polymer-coated urea-N to urea-N at 9:1 (as integrated management 5, T5); Ratio of polymer-coated urea-N to urea-N at 8:2 (as integrated management 6, T6); Ratio of polymer-coated urea-N to urea-N at 7:3 (as integrated management 7, T7); Ratio of polymer-coated urea-N to urea-N at 6:4 (as integrated management 8, T8).

Compared to other treatments, the T7 treatment had the higher yield and crude protein content of silage maize, as shown in **Figure 3**. Treatment 1 without nitrogen fertilizer had the lowest yield at 46.2 t ha^-1^, which was significantly lower by 27.1%-36.7% than that of other fertilization treatments. T3 and T4 did not show a significant decrease in yield compared to T2, even with a reduced nitrogen application of 80 kg ha^-1^. Under the total nitrogen application rate at 240 kg ha^-1^, the T7 treatment achieved the highest yield at 73.1 t ha^-1^, which significantly higher than the other treatments by 6.1% to 58.1%. Furthermore, T7 also had the highest crude protein content at 11.1% (**Figure 3B**), the highest starch content at 19.1% (**Figure 3C**), the lowest acid detergent fiber at 25.9% (**Figure 3D**) and the lowest neutral detergent fiber at 50.6% (**Figure 3E**).

### 3.3 Effect of integrated management strategies on nitrogen use efficiency

As the proportion of polymer-coated urea-N was reduced, the plant nitrogen uptake, nitrogen use efficiency (NUE), nitrogen partial factor productivity (PFP_N_), and nitrogen agronomic efficiency (NAE) exhibited an initial increase followed by a decrease, as detailed in **Table 1**. The T7 treatment displayed the highest values in all these parameters. Specifically, the plant nitrogen uptake reached 200.5 kg ha^-1^, increased by 4.9%-51.7% compared to T1-T8, and its NUE was at 28.4%, PFP_N_ and NAE were recorded at 58.8% and 18.2 kg kg^-1^, respectively. These figures underscore T7’s superior performance in terms of nitrogen utilization efficiency.

**Table 1:** Effect of different management strategies on nitrogen use efficiency of silage maize.

Treatment	Plant nitrogen uptake (kg ha^-1^)	Nitrogen use efficiency (%)	Partial factor productivity of N fertilizer (kg kg^-1^)	Nitrogen agronomic efficiency (kg kg^-1^)
T1	132.2 ± 5.14 c	–	–	–
T2	178.9 ± 4.58 b	14.6 ± 1.81 b	39.9 ± 0.57 c	9.5 ± 0.57 c
T3	183.0 ± 3.35 b	21.1 ± 1.16 ab	54.0 ± 0.93 b	13.4 ± 0.93 b
T4	180.7 ± 2.42 b	20.2 ± 2.69 ab	53.5 ± 0.39 b	12.9 ± 0.39 b
T5	186.4 ± 2.62 ab	22.6 ± 3.20 ab	54.2 ± 0.17 b	13.7 ± 0.17 b
T6	191.2 ± 3.68 ab	24.6 ± 2.31 a	56.7 ± 2.51 ab	16.1 ± 2.51 ab
T7	200.5 ± 10.72 a	28.4 ± 2.35 a	58.8 ± 3.51 a	18.2 ± 3.51 a
T8	182.0 ± 2.64 b	20.7 ± 2.67 ab	54.3 ± 0.80 b	13.7 ± 0.80 b

Different lowercase letters indicate the significant differences at the level of 0.05. No application of nitrogen fertilizer (as control, T1); Applying urea-N 320 kg ha^-1^ according with that in farmers’ practice (as integrated management 2, T2); Applying urea-N 240 kg ha^-1^ (as integrated management 3, T3); Applying polymer-coated urea-N 240 kg N ha^-1^ (as integrated management 4, T4); Ratio of polymer-coated urea-N to urea-N at 9:1 (as integrated management 5, T5); Ratio of polymer-coated urea-N to urea-N at 8:2 (as integrated management 6, T6); Ratio of polymer-coated urea-N to urea-N at 7:3 (as integrated management 7, T7); Ratio of polymer-coated urea-N to urea-N at 6:4 (as integrated management 8, T8).

### 3.4 Effect of integrated management strategies on soil nitrate nitrogen accumulation

**Figure 4:**
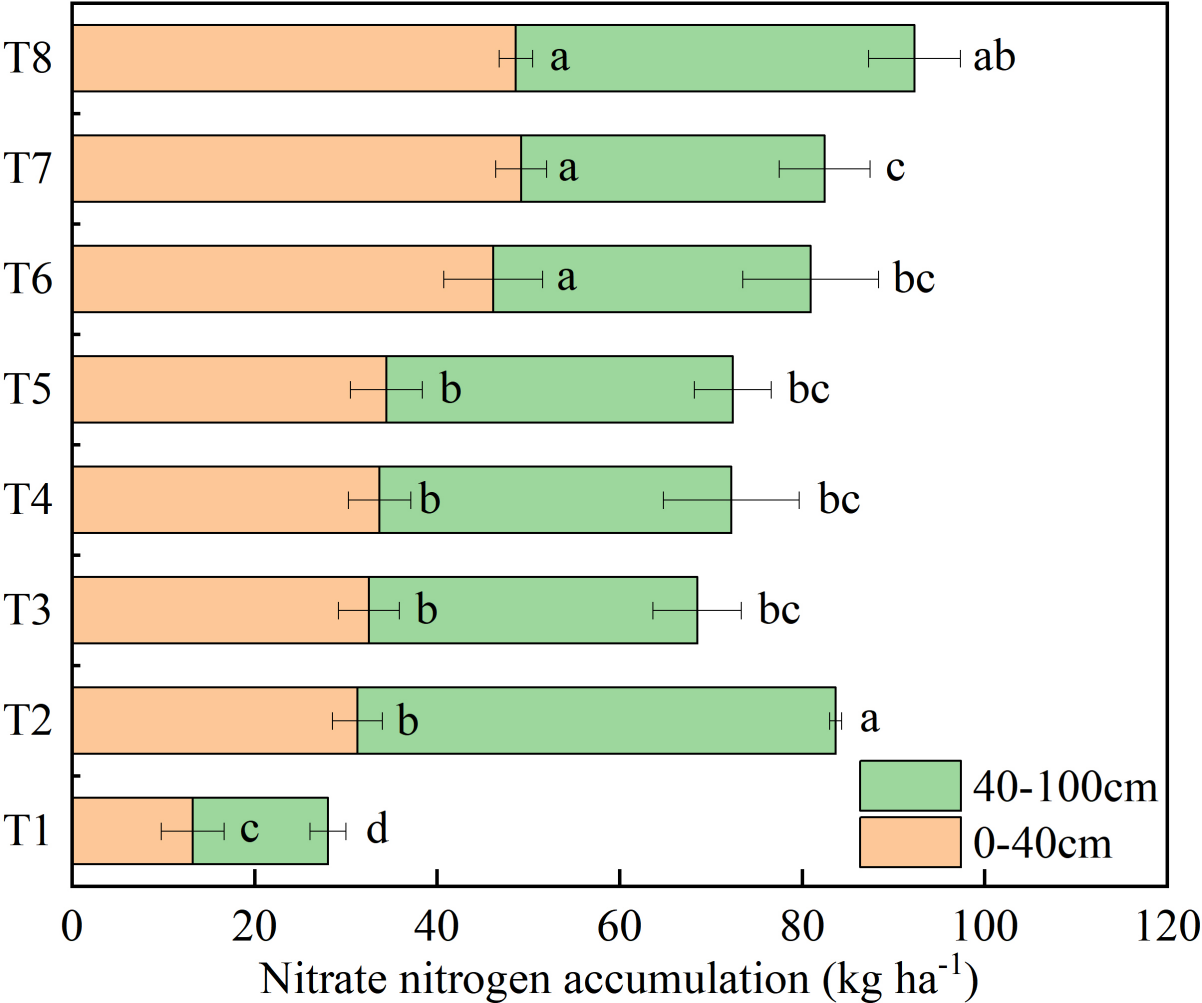
Effect of different management strategies on soil nitrate nitrogen accumulation. Different lowercase letters indicate the significant differences among different treatments in the same soil layer at the level of 0.05. No application of nitrogen fertilizer (as control, T1); Applying urea-N 320 kg ha^-1^ according with that in farmers’ practice (as integrated management 2, T2); Applying urea-N 240 kg ha^-1^ (as integrated management 3, T3); Applying polymer-coated urea-N 240 kg N ha^-1^ (as integrated management 4, T4); Ratio of polymer-coated urea-N to urea-N at 9:1 (as integrated management 5, T5); Ratio of polymer-coated urea-N to urea-N at 8:2 (as integrated management 6, T6); Ratio of polymer-coated urea-N to urea-N at 7:3 (as integrated management 7, T7); Ratio of polymer-coated urea-N to urea-N at 6:4 (as integrated management 8, T8).

Significant differences were observed in the accumulation of nitrate nitrogen among different soil layers and treatments. Compared to the unfertilized control (T1), all fertilizer treatments significantly increased the accumulation of nitrate nitrogen in the 0-100 cm soil layer. However, different optimized nitrogen management strategies resulted in a reduction of nitrate nitrogen accumulation in the deeper soil layer of 40-100 cm, as indicated in **Figure 4**. Notably, even with the nitrogen application rate of 320 kg ha^-1^ in T2, there was no significant difference in nitrate nitrogen accumulation in the 0-40 cm layer compared to T3, T4, and T5, where nitrogen was reduced by 80 kg ha^-1^. In the 0-40 cm layer, the accumulation of nitrate nitrogen in T6, T7, and T8 was significantly higher than that in other treatments, but the differences among these three were not significant. In the 40-100 cm soil layer, T2 exhibited the highest accumulation of nitrate nitrogen at 52.4 kg ha^-1^. However, there was no significant difference among T3 to T7 treatments. Importantly, T7 showed the lowest accumulation of nitrate nitrogen (33.3 kg ha^-1^), effectively reducing the risk of nitrate leaching.

### 3.5 Nitrogen balance

Chemical nitrogen fertilizer is the predominant nitrogen input in agricultural fields, contributing to more than 60% of the total nitrogen input (**Table 2**). Among all evaluated fertilizer treatments, T7 had the lowest nitrogen surplus at 115.1 kg ha^-1^, which was 7.0% to 46.6% lower than that of the other fertilizer treatments. Additionally, T7 also had the highest total nitrogen output, recorded at 283.0 kg ha^-1^. This output was 3.2% to 12.5% higher compared to the other fertilization treatments, underscoring its efficiency in nitrogen utilization.

**Table 2:** Effect of different management strategies on nitrogen balance in soil-maize system (kg ha^-1^).

Item		T1	T2	T3	T4	T5	T6	T7	T8
Input	Chemical N	0	320	240	240	240	240	240	240
	Irrigation water N	4.8	4.8	4.8	4.8	4.8	4.8	4.8	4.8
	Atmospheric N	21.1	21.1	21.1	21.1	21.1	21.1	21.1	21.1
	Mineralized N	132.2	132.2	132.2	132.2	132.2	132.2	132.2	132.2
Output	Plant remove N	132.2	178.9	183.0	180.7	186.4	191.2	200.5	182.0
	0-40 cm NO_3_^–^N	28.0	83.7	68.5	72.3	72.4	81.0	82.5	92.3
Total input		158.1	478.1	398.1	398.1	398.1	398.1	398.1	398.1
Total output		160.2	262.6	251.5	253.0	258.8	272.2	283.0	274.3
Nitrogen surplus		-2.1 e	215.5 a	146.6 b	145.1 b	139.3 bc	125.9 cd	115.1 d	123.8 d

Different lowercase letters indicate the significant differences at the level of 0.05. No application of nitrogen fertilizer (as control, T1); Applying urea-N 320 kg ha^-1^ according with that in farmers’ practice (as integrated management 2, T2); Applying urea-N 240 kg ha^-1^ (as integrated management 3, T3); Applying polymer-coated urea-N 240 kg N ha^-1^ (as integrated management 4, T4); Ratio of polymer-coated urea-N to urea-N at 9:1 (as integrated management 5, T5); Ratio of polymer-coated urea-N to urea-N at 8:2 (as integrated management 6, T6); Ratio of polymer-coated urea-N to urea-N at 7:3 (as integrated management 7, T7); Ratio of polymer-coated urea-N to urea-N at 6:4 (as integrated management 8, T8).

### 3.6 Economic benefits of integrated management strategies

The T7 treatment demonstrated significantly higher economic benefits compared to the other treatments (**Table 3**). In terms of output value, T7 achieved the highest value at 5102 $ ha^-1^, which was significantly greater than that in other treatments (T1-T5) by 8.5% to 58.1%. However, there was no significant difference in output value among T2, T3, T4, and T5. In the aspect of economic benefit, T1 had the lowest net income at 1565 $ ha^-1^, whereas T7 had the highest net income at 3094 $ ha^-1^, and the net incomes among T2, T3, and T4 treatments did not show significant differences. Among the treatments with different ratios of polymer-coated urea-N to urea-N (T5-T8), the fertilizer cost for T7 and T8 was comparatively lower.

**Table 3:** Effect of different management strategies on the economic benefit of silage maize ($ ha^-1^).

Treatment	Fertilizer cost	Other costs	Output value	Economic benefit
T1	197	1466	3228 ± 165 c	1565 ± 165 d
T2	508	1466	4582 ± 303 b	2608 ± 303 bc
T3	430	1466	4702 ± 151 b	2806 ± 151 ab
T4	590	1466	4524 ± 159 b	2468 ± 159 bc
T5	574	1466	4430 ± 250 b	2390 ± 250 c
T6	558	1466	4807 ± 189 ab	2783 ± 189 abc
T7	542	1466	5102 ± 183 a	3094 ± 183 a
T8	526	1466	4770 ± 254 ab	2779 ± 254 abc

Different lowercase letters indicate the significant differences at the level of 0.05. No application of nitrogen fertilizer (as control, T1); Applying urea-N 320 kg ha^-1^ according with that in farmers’ practice (as integrated management 2, T2); Applying urea-N 240 kg ha^-1^ (as integrated management 3, T3); Applying polymer-coated urea-N 240 kg N ha^-1^ (as integrated management 4, T4); Ratio of polymer-coated urea-N to urea-N at 9:1 (as integrated management 5, T5); Ratio of polymer-coated urea-N to urea-N at 8:2 (as integrated management 6, T6); Ratio of polymer-coated urea-N to urea-N at 7:3 (as integrated management 7, T7); Ratio of polymer-coated urea-N to urea-N at 6:4 (as integrated management 8, T8).

## 4 Discussion

### 4.1 Effect of integrated management strategies on yield and quality of silage maize

Selecting excellent varieties, optimizing planting density and applying optimized fertilizer management are necessary measures for improving the quality and yield of maize (Cardwell, 1982; Hu et al., 2013; Zhang et al., 2014). Our results demonstrated that the integrated management strategy 7 had the highest silage maize yield (73.1 t ha^-1^), with an increase of 6.1%-58.1% compared to other treatments (**Figure 3A**). The primary reason for this yield increase is that favorable plant traits promote the biomass formation (Dong et al., 2023). In this study, Xunqing 858 exhibited a distinct advantage in plant height and leaf area. Its plant height was significantly higher than other varieties by 8.7%-22.6%, and its leaf area was significantly higher than Xuntian 3171 by 30.9% (**Figures 2B, D**). This may be attributed to the strong adaptability and stress resistance of Xunqing 858 under unique climatic conditions characterized by low temperature and a short frost-free period. Additionally, a suitable planting density created a favorable population structure (Borrás et al., 2003; Assefa et al., 2016; Piao et al., 2016), enhanced crop light utilization, and ultimately coordinated individual and population yield (Li et al., 2019). The integrated management strategies for silage maize not only affected yield but also impacted its nutritional value. This study found that the integrated management strategy 7 increased crude protein and starch content by 0.2%-21.5% and 0.6%-7.3% (**Figures 3B, C**) compared to other treatments, respectively. This is mainly due to the optimized treatment increasing nitrate nitrogen accumulation in 0-40 cm soil layer (**Figure 4**) and effectively increasing plant nitrogen uptake by 4.9%-51.7% (**Table 1**). These factors promoted the accumulation of enzymatic protein and photosynthesis in plants, leading to quality improvements (Wang et al., 2012; Ma et al., 2013; Zhou et al., 2016).

### 4.2 Effect of integrated management strategies on nitrogen use efficiency and nitrogen balance

Poor management on water and fertilizer during the silage maize production not only leads to the wastage of nutrient resources but also contributes to nitrogen losses and decreases the nitrogen use efficiency (Kang et al., 2016; Srivastava et al., 2020; Wang et al., 2021; Zhao et al., 2022). In recent years, improving nitrogen use efficiency by regulating or altering the transformation or release characteristics of nitrogen fertilizer applied to soil has become a prominent research topic domestically and internationally (Sim et al., 2021). However, the nutrient transformation and release characteristics of commonly used slow-released fertilizers are affected by soil temperature, humidity and other factors (Tlustos and Blackmer, 1992; Husby et al., 2003; Ransom et al., 2020; Lin et al., 2021). Previous studies had shown that the integrated management strategies have a positive impact on nitrogen use efficiency and environmental effects in the plant-soil system (Wang et al., 2020; Chen et al., 2021). Similar results were founded in this study. During the harvest of silage maize, the nitrogen surplus in T7 decreased by 7.0-46.6% (**Table 2**) compared to other management strategies. The main reason was that, under the condition of equal total nitrogen rate of input items, the nitrogen taken up by plants and nitrate nitrogen in 0-40 cm soil were higher in the nitrogen balance output in integrated management strategy 7. Simultaneously, through effectively regulating the soil nitrogen supply and increasing the nitrate accumulation in 0-40 cm soil, the reasonable ratio of polymer-coated urea-N to urea-N in the integrated management strategy 7 (7:3) met the sustained nitrogen demand during the early and later growth stages of silage maize, resulting in higher nitrogen use efficiency. Compared to other treatments, the nitrogen use efficiency, partial factor productivity of N fertilizer and nitrogen agronomic efficiency of T7 increased by 15.4%-94.5%, 3.7%-47.4% and 13.0%-91.6%, respectively (**Table 1**).

### 4.3 Application opportunities and barriers of integrated management strategy

**Figure 5:**
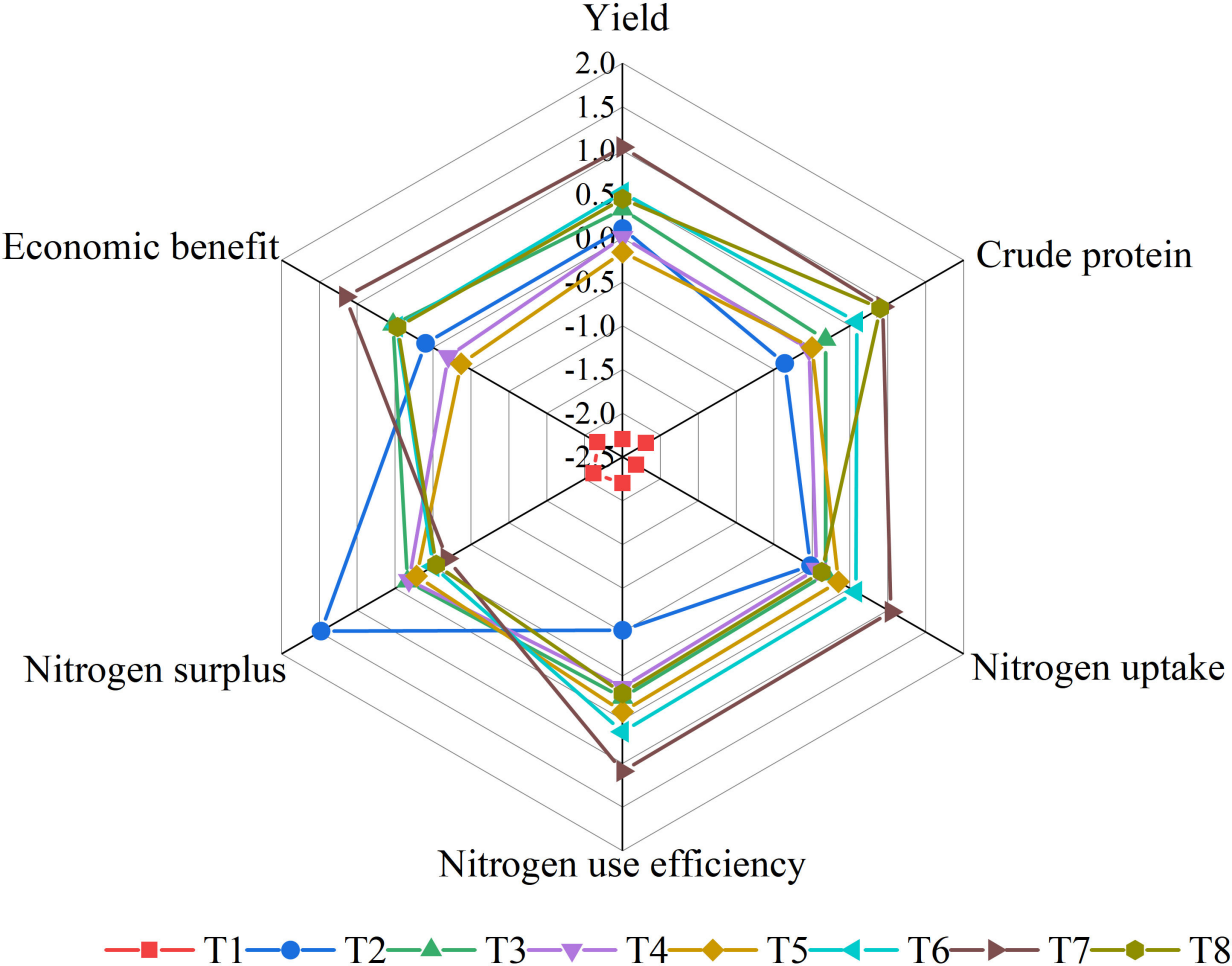
Comprehensive evaluation of different management strategies. No application of nitrogen fertilizer (as control, T1); Applying urea-N 320 kg ha^-1^ according with that in farmers’ practice (as integrated management 2, T2); Applying urea-N 240 kg ha^-1^ (as integrated management 3, T3); Applying polymer-coated urea-N 240 kg N ha^-1^ (as integrated management 4, T4); Ratio of polymer-coated urea-N to urea-N at 9:1 (as integrated management 5, T5); Ratio of polymer-coated urea-N to urea-N at 8:2 (as integrated management 6, T6); Ratio of polymer-coated urea-N to urea-N at 7:3 (as integrated management 7, T7); Ratio of polymer-coated urea-N to urea-N at 6:4 (as integrated management 8, T8).

Against the backdrop of increasing pressure on feed supply and environmental resources, achieving further improvement in silage maize yield and quality, as well as environmental protection, poses significant challenges in the development of livestock industry in China (Hu et al., 2019; Randby et al., 2019). Integrated management strategies, through optimizing crop varieties and densities and employing a balanced combination of polymer-coated urea-N to urea-N, provide a practical approach to address the issues of low and unstable yield and quality, as well as nitrogen excess in silage maize production in cold regions, while reducing environmental pollution (**Figure 5**). However, there are still many obstacles and challenges to applying and promoting these management strategies. Firstly, in terms of personnel allocation, China’s agriculture is primarily operated by millions of small-scale farmers, which poses a significant challenge to the widespread implementation of integrated management technologies (Spielman et al., 2010; Cui et al., 2018; Yin et al., 2019; Zhang et al., 2020). Secondly, in terms of the controllability of technology, rural areas, especially in cold regions, are sparsely populated and unattractive to young generations, making it difficult to adopt new technologies or production transformations (Zhang et al., 2020). Lastly, in terms of economic factors, technology cost is an important driving factor in determining the implementation of this technology (Dreher et al., 2003). Due to the high labor cost of fertilization, farmers are increasingly inclined to apply all fertilizers before sowing (without top dressing). Hence, farmers have a great demand for slow-released compound fertilizers (Li et al., 2022). However, the technology recommended in this study requires a reasonable proportion of polymer-coated urea-N to urea-N, which increases the difficulty of popularization.

To implement this effective technology strategy, we should (1) increase technical services to enable farmers to adapt and adopt more knowledge-intensive agricultural practices (Chen et al., 2014); (2) increase institutional support and infrastructure construction, and transfer integrated management technologies to millions of small-scale farmers (Zhang et al., 2016). (3) Governments should increase policy support and financial investment in the agricultural sector. Importantly, farmers must receive proper remuneration for using this technology. Additionally, the new technology must be evaluated and receive feedback for end-user, including farmers. Also, the technology can then be further improved and better adapted to the specific agricultural conditions relevant to the end-users, reflecting a bidirectional feedback mechanism via iteration (Shen et al., 2024; Zhang et al., 2024).

In summary, concerted efforts from multiple stakeholders are required to conduct sufficient localized studies to achieve widespread application and promotion of these management strategies globally. This is paramount for realizing the green development of livestock industry. The integrated management technology was studied in cold regions in China. However, this idea and the related results can also provide important references for silage maize production in similar regions of other countries. It is only necessary to update management strategies according to local climate and soil properties to promote the sustainable development of the global livestock industry.

## 5 Conclusion

The integrated management strategy 7, which optimized silage maize variety Xunqing 858 with a planting density of 79,500 plants ha^-1^, and applied 240 kg N ha^-1^ with a nitrogen formula ratio of 7:3 (polymer-coated urea-N to urea-N), can effectively increase yield and quality, improve nitrogen use efficiency and economic benefits, as well as reduce soil nitrogen surplus in the silage maize production system in cold regions. However, we acknowledge that there are still planting systematic, technological, personnel and financial barriers to implementing this integrated management strategy, which requires concerted efforts from multiple stakeholders. Nevertheless, the successful application of this strategy will provide valuable insights for promoting the sustainable development of livestock husbandry facing similar challenges worldwide.

## Data availability statement

The original contributions presented in the study are included in the article/supplementary material. Further inquiries can be directed to the corresponding authors.

## Author contributions

CL: Formal analysis, Writing – original draft. BT: Writing – review & editing. MJ: Methodology, Writing – original draft. HX: Writing – review & editing. JW: Conceptualization, Funding acquisition, Investigation, Resources, Writing – review & editing. ZS: Conceptualization, Funding acquisition, Investigation, Resources, Writing – review & editing.

## References

[B1] Assefa Y. Carter P. Hinds M. Bhalla G. Schon R. Jeschke M.. (2018). Analysis of long term study indicates both agronomic optimal plant density and increase maize yield per plant contributed to yield gain. Sci. Rep. 8, 4937. doi: 10.1038/s41598-018-23362-x 29563534 PMC5862987

[B2] Assefa Y. Prasad P. V. V. Carter P. Hinds M. Bhalla G. Schon R.. (2016). Yield responses to planting density for US modern corn hybrids: a synthesis-analysis. Crop Science. 56, 2802–2817. doi: 10.2135/cropsci2016.04.0215

[B3] Bai L. F. Zhang X. Q. Li B. Z. Sun F. C. Zhao X. Q. Wang Y. F.. (2022). Fungal communities are more sensitive to nitrogen fertilization than bacteria in different spatial structures of silage maize under short-term nitrogen fertilization. Appl. Soil Ecology. 170, 104275. doi: 10.1016/j.apsoil.2021.104275

[B4] Bao S. D. (2000). Soil agro-chemistrical analysis (Beijing: China Agriculture Press).

[B5] Bilal A. K. Adnan M. Rehman F. U. Hasnain A. Usman M. Javed M. S.. (2021). Role of silage in agriculture: A review. Green Rep. 2, 9–12. doi: 10.36686/Ariviyal.GR.2021.02.04.010

[B6] Borrás L. Maddonni G. A. Otegui M. E. (2003). Leaf senescence in maize hybrids: Plant population, row spacing and kernel set effects. Field Crops Res. 82, 13–26. doi: 10.1016/S0378-4290(03)00002-9

[B7] Cardwell V. B. (1982). Fifty years of Minnesota corn production: sources of yield increase. Agron. J. 74, 984–990. doi: 10.2134/agronj1982.00021962007400060013x

[B8] Chen L. Xie H. Wang G. L. Yuan L. M. Qian X. Q. Wang W. L.. (2021). Reducing environmental risk by improving crop management practices at high crop yield levels. Field Crops Res. 265, 108123. doi: 10.1016/j.fcr.2021.108123

[B9] Chen X. P. Cui Z. L. Fan M. S. Vitousek P. Zhao M. Ma W. Q.. (2014). Producing more grain with lower environmental costs. Nature. 514, 486–489. doi: 10.1038/nature13609 25186728

[B10] Chen X. P. Cui Z. L. Vitousek P. M. Cassman K. G. Matson P. A. Bai J. S.. (2011). Integrated soil-crop system management for food security. Proc. Natl. Acad. Sci. United States America. 108, 6399–6404. doi: 10.1073/pnas.1101419108 PMC3080987 21444818

[B11] China Statistical Yearbook (2014, 2023). National Bureau of Statistics of China. Beijing: National Bureau of Statistics of China. in Chinese.

[B12] Cui Z. L. Zhang H. Y. Chen X. P. Zhang C. C. Ma W. Q. Huang C. D.. (2018). Pursuing sustainable productivity with millions of smallholder farmers. Nature. 555, 363–366. doi: 10.1038/nature25785 29513654

[B13] Dong Y. Li X. Y. Yan F. Zhao F. Y. Hou X. M. Zhao X. M.. (2023). Comprehensive evaluation of 12 silage maize varietiesin qiqihar area. Seed. 42, 68–72. doi: 10.16590/j.cnki.1001-4705.2023.09.068

[B14] Dreher K. Khairallah M. Ribaut J. M. Morris M. (2003). Money matters (I): costs of field and laboratory procedures associated with conventional and marker-assisted maize breeding at CIMMYT. Mol. Breeding. 11, 221–234. doi: 10.1023/A:1022820520673

[B15] Du Z. M. Yang F. Y. Fang J. C. Cai Y. M. Oya T. Nguluve D.. (2023). Silage preparation and sustainable livestock production of natural woody plant. Front. Plant Science. 14. doi: 10.3389/fpls.2023.1253178 PMC10514673 37746011

[B16] Duvick D. N. (2005). The contribution of breeding to yield advances in maize (*Zea mays L.*). Adv. Agronomy. 86, 83–145. doi: 10.1016/S0065-2113(05)86002-X

[B17] Fallah S. Tadayyon A. (2010). Uptake and nitrogen efficiency in forage maize: effects of nitrogen and plant density. Agrociencia. 44, 549–560. doi: 10.1016/j.agsy.2010.03.012

[B18] Galeano E. S. J. Costa C. M. Orrico M. A. P. Fernandes T. Retore M. Silva M. S. J.. (2021). Agronomic aspects, chemical composition and digestibility of forage from corn-crotalaria intercropping. J. Agric. Science. 159, 580–588. doi: 10.1017/S0021859621000848

[B19] Guyader J. Baron V. S. Beauchemin K. A. (2018). Corn forage yield and quality for silage in short growing season areas of the Canadian prairies. Agron. Basel. 8, 164. doi: 10.3390/agronomy8090164

[B20] Hu C. L. Sadras V. O. Lu G. Y. Jin X. Xu J. X. Ye Y. L.. (2019). Dual-purpose winter wheat: interactions between crop management, availability of nitrogen and weather conditions. Field Crops Res. 241, 107579. doi: 10.1016/j.fcr.2019.107579

[B21] Hu H. Y. Ning T. Y. Li Z. J. Han H. F. Zhang Z. Z. Qin S. J.. (2013). Coupling effects of urea types and subsoiling on nitrogen-water use and yield of different varieties of maize in northern China. Field Crops Res. 142, 85–94. doi: 10.1016/j.fcr.2012.12.001

[B22] Husby C. E. Niemiera A. X. Harris J. R. Wright R. D. (2003). Influence of diurnal temperature on nutrient release patterns of three polymer-coated fertilizers. HortScience. 38, 387–389. doi: 10.21273/HORTSCI.38.3.387

[B23] Ju X. T. Kou C. L. Zhang F. S. Christie P. (2006). Nitrogen balance and groundwater nitrate contamination: Comparison among three intensive cropping systems on the North China Plain. Environ. Pollution. 143, 117–125. doi: 10.1016/j.envpol.2005.11.005 16364521

[B24] Kang Y. Liu M. Song Y. Huang X. Yao H. Cai X. H.. (2016). High-resolution ammonia emissions inventories in China from 1980 to 2012. Atmospheric Chem. Physics. 16, 2043–2058. doi: 10.5194/acp-16-2043-2016

[B25] Komarek A. M. Dunston S. Enahoro D. Godfray H. C. J. Herrero M. Mason-D'Croz D.. (2021). Income, consumer preferences, and the future of livestock-derived food demand. Global Environ. Change. 70, 102343. doi: 10.1016/j.gloenvcha.2021.102343 PMC7612057 34857999

[B26] Kumar U. Halling M. Parsons D. Bergkvist G. Morel J. Vogeler I.. (2022). Dynamics and plasticity of agronomic performance and nutritive quality traits in forage maize at high latitudes. Eur. J. Agronomy. 138, 126532. doi: 10.1016/j.eja.2022.126532

[B27] Li N. Yang Y. Wu Y. J. Liu B. M. Tao L. Z. Zhan Y.. (2022). Better performance of compound fertilizers than bulk-blend fertilizers on reducing ammonia emission and improving wheat productivity. Agric. Ecosyst. Environment. 335, 108018. doi: 10.1016/j.agee.2022.108018

[B28] Li R. F. Liu P. Dong S. T. Zhang J. W. Zhao B. (2019). Increased maize plant population induced leaf senescence, suppressed root growth, nitrogen uptake, and grain yield. Agron. J. 111, 1581–1591. doi: 10.2134/agronj2018.09.0554

[B29] Lin X. Y. Guo L. Z. Shaghaleh H. Hamoud Y. A. Xu X. Liu H. (2021). A TEMPO-oxidized cellulose nanofibers/MOFs hydrogel with temperature and pH responsiveness for fertilizers slow-release. Int. J. Biol. Macromolecules. 191, 483–491. doi: 10.1016/j.ijbiomac.2021.09.075 34562535

[B30] Liu G. Z. Yang H. S. Xie R. Z. Yang Y. S. Liu W. M. Guo X. X.. (2021). Genetic gains in maize yield and related traits for high-yielding cultivars released during 1980s to 2010s in China. Field Crops Res. 270, 108223. doi: 10.1016/j.fcr.2021.108223

[B31] Liu X. J. Zhang Y. Han W. X. Tang A. H. Shen J. L. Cui Z. L.. (2013). Enhanced nitrogen deposition over China. Nature. 494, 459–462. doi: 10.1038/nature11917 23426264

[B32] Liu Z. Gao J. Gao F. Dong S. T. Liu P. Zhao B.. (2018). Integrated agronomic practices management improve yield and nitrogen balance in double cropping of winter wheat-summer maize. Field Crops Res. 221, 196–206. doi: 10.1016/j.fcr.2018.03.001

[B33] Ma L. Yuan F. Zhu L. L. Wang Z. M. Rong Y. P. (2013). Yield and quality of silage corn (Zea mays) as affected by type and quantity of N fertilization. Acta Prataculturae Sinica. 22, 53–59. doi: 10.11686/cyxb20130607

[B34] Niu Y. J. Zhu H. M. Yang S. W. Ma S. J. Zhou J. W. Chu B.. (2019). Overgrazing leads to soil cracking that later triggers the severe degradation of alpine meadows on the Tibetan Plateau. Land Degradation Dev. 30, 1243–1257. doi: 10.1002/ldr.3312

[B35] Piao L. Qi H. Li C. F. Zhao M. (2016). Optimized tillage practices and row spacing to improve grain yield and matter transport efficiency in intensive spring maize. Field Crops Res. 198, 258–268. doi: 10.1016/j.fcr.2016.08.012

[B36] Randby A. T. Nadeau E. Karlsson L. Johansen A. (2019). Effect of maturity stage at harvest and kernel processing of whole crop wheat silage on digestibility by dairy cows. Anim. Feed Sci. Technology. 253, 141–152. doi: 10.1016/j.anifeedsci.2019.04.016

[B37] Ransom C. J. Jolley V. D. Blair T. A. Sutton L. E. Hopkins B. G. (2020). Nitrogen release rates from slow- and controlled-release fertilizers influenced by placement and temperature. PloS One 15, e0234544. doi: 10.1371/journal.pone.0234544 32555670 PMC7299380

[B38] Ru S. H. Zhang G. Y. Sun S. Y. Wang L. Geng N. (2013). Status of the contamination and spatial-temporal variations of nitrate in groundwater of Hebei Province, China. J. Agric. Resour. Environment. 30, 48–52. doi: 10.13254/j.jare.2013.05.010

[B39] Sezer I. Kiremit M. S. Öztürk E. Subrata B. A. G. Osman H. M. Akay H.. (2021). Role of melatonin in improving leaf mineral content and growth of sweet corn seedlings under different soil salinity levels. Scientia Horticulturae. 288, 110376. doi: 10.1016/j.scienta.2021.110376

[B40] Shen J. B. Zhu Q. C. Hou Y. Cong W. F. Xu W. Xu J. L.. (2024). Agriculture green development in China: insights and advances. Front. Agric. Sci. Engineering. 11, 5–19. doi: 10.15302/J-FASE-2024535

[B41] Sim D. H. H. Tan I. A. W. Lim L. L. P. Hameed B. H. (2021). Encapsulated biochar-based sustained release fertilizer for precision agriculture: A review. J. Cleaner Production. 303, 127018. doi: 10.1016/j.jclepro.2021.127018

[B42] Spielman D. J. Byerlee D. Alemu D. Kelemework D. (2010). Policies to promote cereal intensifcation in Ethiopia: the search for appropriate public and private roles. Food Policy. 35, 185–194. doi: 10.1016/j.foodpol.2009.12.002

[B43] Srivastava R. K. Panda R. K. Chakraborty A. (2020). Quantification of nitrogen transformation and leaching response to agronomic management for maize crop under rainfed and irrigated condition. Environ. Pollution. 265, 114866. doi: 10.1016/j.envpol.2020.114866 32505935

[B44] Tlustos P. Blackmer A. M. (1992). Release of nitrogen from ureaform fractions as influenced by soil pH. Soil Sci. Soc. America J. 56, 1807–1810. doi: 10.2136/sssaj1992.03615995005600060026x

[B45] Wang P. Zhao Z. Y. Wang L. Tian C. Y. (2021). Comparison of efficiency-enhanced management and traditional management of irrigation and nitrogen fertilization in cotton fields of northwestern China. Agriculture-Basel. 11, 1134. doi: 10.3390/agriculture11111134

[B46] Wang X. W. Ma X. Zhou L. R. Xia Y. Dai J. J. (2012). Effect of nitrogen fertilization on corn yield, nitrogen accumulation and physiology index. J. Maize Sci. 20, 121–125. doi: 10.13597/j.cnki.maize.science.2012.05.027

[B47] Wang X. Z. Zhao M. J. Liu B. Zou C. Q. Sun Y. X. Wu G.. (2020). Integrated systematic approach increase greenhouse tomato yield and reduce environmental losses. J. Environ. Management. 266, 110569. doi: 10.1016/j.jenvman.2020.110569 32310118

[B48] Wheeler T. von Braun J. (2013). Climate change impacts on global food security. Science. 341, 508–513. doi: 10.1126/science.1239402 23908229

[B49] Wu G. Yang S. Luan C. S. Wu Q. Lin L. L. Li X. X.. (2024). Partial organic substitution for synthetic fertilizer improves soil fertility and crop yields while mitigating N_2_O emissions in wheat-maize rotation system. Eur. J. Agronomy. 154, 127077. doi: 10.1016/j.eja.2023.127077

[B50] Yang J. Hou L. Y. Bai W. M. Yan J. Y. Hao J. X. Tao J.. (2019). A dual-purpose model for spring-sown oats in cold regions of northern China. Agron. Basel. 9, 721. doi: 10.3390/agronomy9110721

[B51] Yin Y. L. Ying H. Xue Y. F. Zheng H. F. Zhang Q. S. Cui Z. L. (2019). Calculating socially optimal nitrogen (N) fertilization rates for sustainable N management in China. Sci. Total Environment. 688, 1162–1171. doi: 10.1016/j.scitotenv.2019.06.398 31726547

[B52] Zeng T. R. Wu Y. S. Xin Y. F. Chen C. Du Z. C. Li X. L.. (2022). Silage quality and output of different maize-soybean strip intercropping patterns. Fermentation Basel. 8, 174. doi: 10.3390/fermentation8040174

[B53] Zhang F. S. Wang J. Q. Zhang W. F. Cui Z. L. Ma W. Q. Chen X. P.. (2008). Nutrient use efficiencies of major cereal crops in China and measures for improvement. Acta Pedologica Sinica. 05), 915–924. doi: 10.3969/j.issn.1003-1650.2013.12.074

[B54] Zhang H. X. Feng Y. Jia Y. X. Liu P. Q. Hou Y. Shen J. B.. (2024). China’s agriculture green development: from concept to actions. Front. Agric. Sci. Engineering. 11, 20–34. doi: 10.15302/J-FASE-2023512

[B55] Zhang Q. Zhang L. Z. Evers J. C. van der Werf W. Zhang W. Q. Duan L. S. (2014). Maize yield and quality in response to plant density and application of a novel plant growth regulator. Field Crops Res. 164, 82–89. doi: 10.1016/j.fcr.2014.06.006

[B56] Zhang Q. S. Chu Y. Y. Xue Y. F. Ying H. Chen X. H. Zhao Y. J.. (2020). Outlook of China’s agriculture transforming from smallholder operation to sustainable production. Global Food Security. 26, 100444. doi: 10.1016/j.gfs.2020.100444

[B57] Zhang W. H. Jia Z. B. Zhuo Y. Jing X. Y. (2016). Space dynamic change of pasture amount and influence factors analysis in Xilin Gol Grassland. J. Earth Environment. 7, 163–172. doi: 10.7515/JEE201602006

[B58] Zhao J. Pullens J. W. M. Sørensen P. Blicher-Mathiesen G. Olesen J. E. Børgesen C. D. (2022). Agronomic and environmental factors influencing the marginal increase in nitrate leaching by adding extra mineral nitrogen fertilizer. Agriculture Ecosyst. Environment. 327, 107808. doi: 10.1016/j.agee.2021.107808

[B59] Zhao Y. N. Huang Y. F. Li S. Chu X. Ye Y. L. (2020). Improving the growth, lodging and yield of different density-resistance maize by optimising planting density and nitrogen fertilisation. Plant Soil Environment. 66, 453–460. doi: 10.17221/178/2020-PSE

[B60] Zhao Z. Wang G. F. Chen J. C. Wang J. Y. Zhang Y. (2019). Assessment of climate change adaptation measures on the income of herders in a pastoral region. J. Cleaner Production. 208, 728–735. doi: 10.1016/j.jclepro.2018.10.088

[B61] Zhou B. Y. Wang X. B. Wang Z. M. Ma W. Zhao M. (2016). Effect of slow-release fertilizer and tillage practice on grain yield and nitrogen efficiency of summer maize (Z.mays L.). J. Plant Nutr. Fertilizer. 22, 821–829. doi: 10.11674/zwyf.14526

